# Standardization of Dental Vulnerability Scale scores (EVO-BR)

**DOI:** 10.1186/s12903-024-04531-z

**Published:** 2024-07-14

**Authors:** Daniele Boina de Oliveira, Lorrayne Belotti, Ilana Eshriqui, Flávio Rebustini, Danielle da Costa Palácio, Daiana Bonfim, Marcio Anderson Cardozo Paresque, Danielle Viana Ribeiro, Wander Barbieri, Tamara Kerber Tedesco

**Affiliations:** 1 Graduate Program in Dentistry, Department of Odontology , Cruzeiro do Sul University, São Paulo (SP), Brazil; 2 Albert Einstein Center for Studies, Research and Practice in Primary Health Care (CEPPAR), Hospital Israelita Albert Einstein, São Paulo (SP), Brazil; 3 Graduate Program in Gerontology, The School of Arts, Sciences and Humanities, São Paulo University, São Paulo (SP), Brazil; 4 Department of Orthodontics and Pediatric Dentistry, School of Dentistry, University of São Paulo, São Paulo (SP), Brazil

**Keywords:** Standardization, Instrument, Vulnerability, Oral health, Public health, Primary care

## Abstract

**Background:**

Dental Vulnerability Scale (EVO-BR) is an instrument developed to help identifying oral health-vulnerable individuals. This scale comprises 15 items distributed into 4 dimensions. It is the first instrument with the potential to guide clinical and managerial decisions in the oral health field. The aim is to validate a score to enable using EVO-BR in Primary Health Care (PHC).

**Method:**

The investigated sample included PHC users in five Brazilian regions. Data were collected at two different stages: in 2019 (São Paulo) and in 2022 (Minas Gerais, Mato Grosso, Roraima, Pernambuco e Paraná). Exploratory descriptive study of this scale scores was carried out to create classification ranges. Subsequently, discriminant analysis was performed to assess the accuracy of the established classification. Boosting regression was carried out to check items’ weight for the instrument score.

**Results:**

EVO-BR score ranged from 0 (highest vulnerability) to 15 (lowest vulnerability). Four (4) classification configurations were tested. Score equal to 12 points was the one presenting the best classification of the assessed individuals (100% were correctly classified). Boosting regression has evidenced that items 1 and 2 (Overall health domain) and 14 and 15 (Health Services domain) had the strongest influence on this instrument’s score.

**Conclusion:**

The process to standardize the EVO-BR score and, consequently, to develop assessment ranges, is an important step in the fight against health inequalities, since it provides a tool to help planning actions and interventions aimed at meeting specific needs of the population in the Primary Health Care context.

## Background

Oral health vulnerability results from the interaction of several factors capable of compromising oral health conditions. These factors result in individuals’ precarious health condition, a fact that makes it hard for them to have access to dental services, mainly socially vulnerable groups [1]. Social, economic, cultural and demographic disparities highlight the solid link between these conditions and oral health. Although some developed contexts provide greater first dental appointment coverage and access to oral health care to the population [2], overall, inequality between oral health care supply and demand at Primary Health Care (PHC) scope persists [3].

Several factors capable of both determining and conditioning individuals’ health play crucial role in either positively or negatively influencing population’s access to dental treatment services. Therefore, it is essential understanding these elements to enable implementing oral health care organization processes in compliance with universality, comprehensiveness and equity principles [4–6]. This understanding enables decision-making associated with implementing public policies focused on meeting population’s real needs [7].

Accordingly, the Dental Vulnerability Scale (EVO-BR), which adopts a comprehensive approach to the concept of vulnerability [8], is a nationally validated instrument used to identify oral health determining and conditioning factors [9]. It comprises 15 items and can be applied by PHC professionals to cover different dimensions in General Health, Oral Health, Infrastructure and Health Services. Its results have the potential to guide oral health teams in effectively planning actions and interventions [10, 11].

Given the previous evidence of EVO-BR validity and its potential applicability as working instrument to help organizing the access to and provision of oral health care, the aim of the current study was to validate a score to enable using EVO-BR in PHC practice.

## Methods

Study focused on standardizing the general scores of the Dental Vulnerability Scale (EVO-BR) [9]. The sample selection method for this study is convenience sampling, applied to users of the Unified Health System aged 18 or over who were present at the 18 units of PHC for consultation with higher education professionals. The total sample was 1753 respondents. The sizing of participants in psychometric studies is usually done based on the number of items [12] that demonstrated a ratio of 20:1 or greater, that is, 20 respondents for each item of the instrument It would be ideal. However, ratios of 10:1 already allow analyzes with low associated errors resulting from the sample size, with a ratio of 5:1 being the minimum acceptable [13].

### Scenario

The herein investigated sample was formed by users of Primary Health Care Units (PHCU) located in different Brazilian geographic regions. Specific criteria were adopted to select the PHCU, namely: 1: PHCU located in municipalities that followed the Health Care Planning methodology were selected to ensure the representativeness of at least one PHCU in each of the five Brazilian regions; 2: priority was given to the most populous municipalities to ensure a comprehensive sample; as well as to 3: those providing easy access for data collectors.

Based on these criteria, EVO-BR was applied in 19 PHCU located in specific municipalities, namely:

- Southeastern region: Uberlândia – MG (2 PHCU) and São Paulo - SP (11 PHCU);
- Southern region: Irati and Teixeira Soares – PR (2 PHCU);
- Northeastern region: Belo Jardim – PE (1 PHCU);
- Central-Western region: Rondonópolis – MT (2 PHCU);
- Northern region: Boa Vista – RR (1 PHCU).

Data collection took place at two different stages: the first one was carried out by dental surgeons in São Paulo, between September and November 2019; whereas the second one was conducted in Minas Gerais, Mato Grosso, Roraima, Pernambuco and Paraná states, between May and August 2022. Data collection conducted at the second stage was carried out by previously calibrated collectors, mainly in municipalities outside São Paulo.

### Study population

The following inclusion criteria were adopted: participants must be healthcare service users; be 18 years old or older; and have attended PHCU during data collection period. Potential participants were approached both in the dental office and in the PHCU waiting room, where they were invited to participate in the study.

After participants were introduced to the research project and provided informed consent by signing the Free and Informed Consent Form (TCLE), they completed a structured questionnaire comprising clinical and sociodemographic information, as well as all 41 EVO items for validation purposes. Research Electronic Data Capture (REDCAP) software was used for data collection and storage purposes [14].

### Data analysis

Results recorded for each item and for the total score were expressed as response frequency, median (Md), interquartile range (IQR), range (amp), minimum (min) and maximum (max). The standardization process identified cut-off points based on participants’ distribution. Discriminant analysis was applied to each range to ensure high accuracy in the proposed cut-off points and to check their predictive ability to classify individuals. This analysis was applied to confirm whether the cut-off points established based on participants’ distribution could properly classify individuals into the proposed ranges [15–17].

Machine learning boosting regression was used with 50% cross validation to identify instrument items, as well as dimensions presenting the highest relative influence on the instrument’s score. Data were analyzed in SPSS v.23 and JASP 17.03 statistical software.

### Ethical aspects

The current study was approved by the Research Ethics Committee of Albert Einstein Israelite Hospital (CAAE: 12395919.0.0000.0071).

## Results

Study population comprised 1,753 participants. Most respondents belonged to the male sex (52.01%) were in the mean age group of 39 years. Race/skin color distribution was similar among white (30.20%), black (34.44%) and brown (32.69%) individuals. According to most participants, the dweller: room ratio in their homes was equal to, or lower than, one (81.42%).

Table 1 presents the absolute and relative frequency of responses to EVO-BR items. The lower the instrument score, the higher participants’ vulnerability. With respect to the analyzed dimensions, the *General Health* block comprised 3 items (items 1 to 3): “1 – Yes” response to these items pointed out vulnerability, whereas potential for vulnerability in the other domains, i.e., in items 4 to 15, lied on response “0 – No” (Table 1). Thus, it was necessary developing a formula for EVO calculation purposes (Eq. 1). Results pointed towards a score range that could go from 0 (the highest vulnerability level) to 15 (the lowest vulnerability level).

**Table 1:** Absolute and relative frequency of responses to EVO-BR items

Dimension	Item	Response Frequency (N/%)		
		No	Yes	Missing
General Health	Does your health prevent you from performing some daily activities?	1.496 (85.3)	256 (14.6)	2 (0.1)
	Do you have any movement impairment?	1.547 (88.2)	205 (11.7)	2 (0.1)
	Do you have any disease requiring monitoring?	1.244 (70.9)	508 (29.0)	2 (0.1)
Oral Health	Do you consider oral care important?	43 (2.5)	1706 (97.3)	5 (0.3)
	Do you believe that oral diseases can be prevented?	20 (1.1)	1728 (98.5)	6 (0.3)
	Do you believe you treat your oral health in a responsible manner?	73 (4.2)	1674 (95.4)	7 (0.4)
	Do you believe it is important to have all teeth in your mouth?	16 (0.9)	1732 (98.7)	6 (0.3)
Infrastructure	Is there a bathroom in your home?	19 (1.1)	1730 (98.6)	5 (0.3)
	Is there electricity in your home?	17 (1.0)	1729 (98.6)	8 (0.5)
	Is there running water in your home?	29 (1.7)	1716 (97.8)	9 (0.5)
	Is there sewage collection in your home?	117 (6.7)	1630 (92.9)	7 (0.4)
Health Care Services	Do you know the public health center where you can seek medical help?	54 (3.1)	1694 (96.6)	6 (0.3)
	Do you use public health center services?	97 (5.5)	1652 (94.2)	5 (0.3)
	Are you monitored by an oral health team?	531 (30.3)	1217 (69.4)	6 (0.3)
	Do you have access to free dental care service?	448 (25.5)	1298 (74.0)	8 (0.5)

1 $${\rm{EVO }} = {\Sigma _{{\rm{items}}\,{\rm{from}}\,{\rm{4 \,to}}\,{\rm{15}}}} + (3-{\Sigma _{{\rm{General}}\,{\rm{Health}}}})$$

Table 2 presents dimension scores and the general EVO-BR score. All domains had their range responded. Total scale score ranged from 3 to 15. The Oral Health and Infrastructure domains had interquartile range equal to zero, and it points out scores’ concentration and no difference between the 25th percentile score and the 75th percentile score. General Health and Health Services just scored 1 point in the interquartile range. The overall score presented range of just 2 points.

**Table 2:** Description of family vulnerability scale dimensions and score

Dimension/Score	Central Trend and Dispersion Measures				
	Median	Minimum	Maximum	Range	Interquartile range
General Health	0.00	0.00	3.00	3.00	1.00
Oral Health	4.00	0.00	4.00	4.00	0.00
Infrastructure	4.00	0.00	4.00	4.00	0.00
Health Care Services	4.00	0.00	4.00	4.00	1.00
**EVO-BR score**	14.00	3.00	15.00	12.00	2.00

**Fig. 1:**
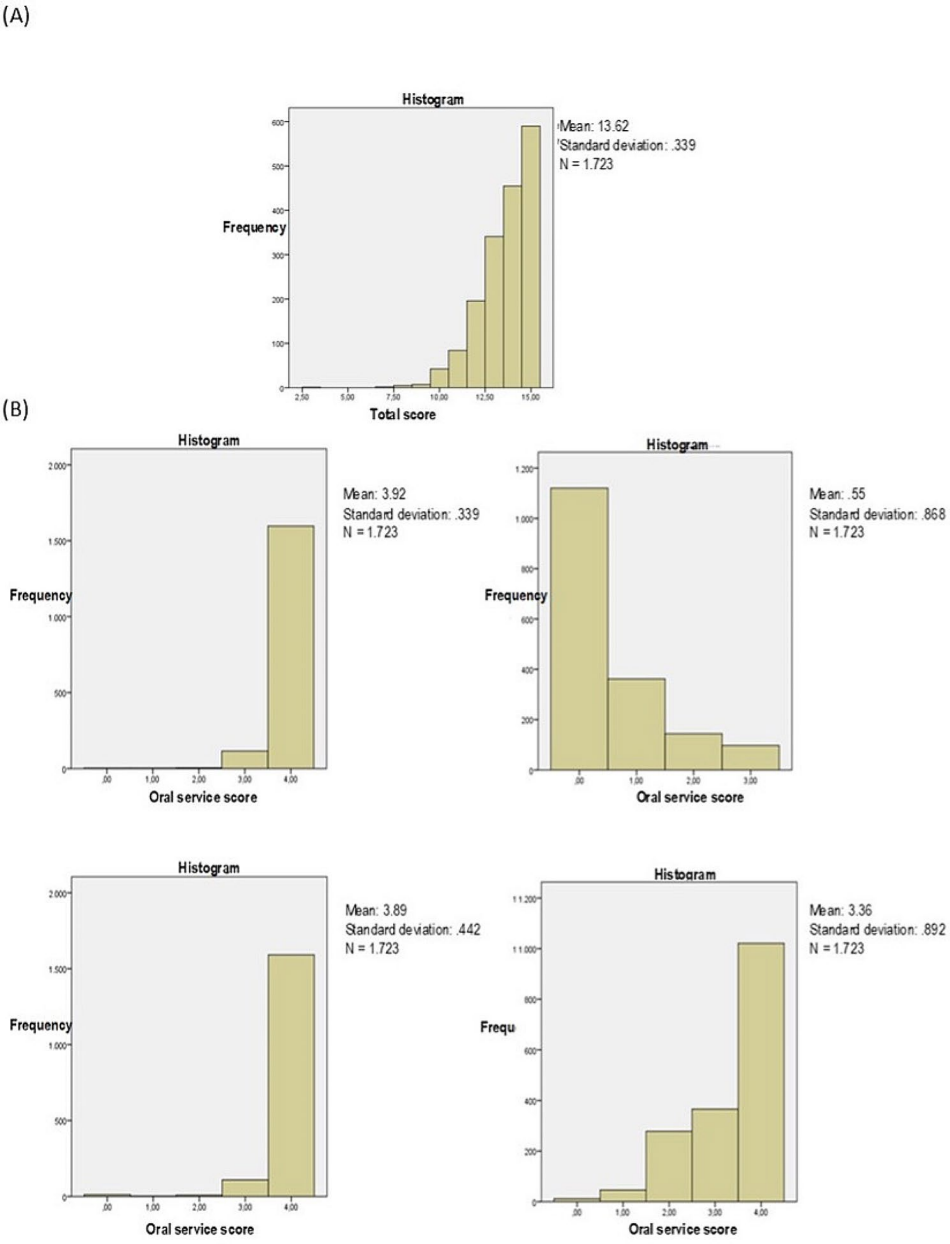
Histogram plotted for EVO-BR (**A**) total score and its dimension (**B**)

EVO-BR score distribution presented strong negative asymmetry (Fig. 1A), and it justified the zero range, whereas the General Health dimension presented positive distribution, inverse to that of the other dimensions. This finding legitimized the development of the calculation formula (Fig. 1B).

As previously mentioned, ranges were very close to each other. Based on Table 3, although the instrument’s general range goes from 0 to 15, 95% of data were higher than, or equal to, score 11. Given this proximity, it was not possible clearly determining individuals’ classification based on just using quartiles. Option was made to conduct discriminant analysis with 4 possible cut-off points, starting from score 12, which represents the 10th percentile, as well as for establishing the cut-off points for scores 11, 10 and 9. This approach only led to two classification ranges, namely: high and low vulnerability (Table 4).

**Table 3:** Percentiles and total score

Percentile	Total score
5th	11.00
10th	12.00
25th	13.00
50th	14.00
75th	15.00
90th	15.00
95th	15.00

**Table 4:** Ranges, classification and interpretation of Dental Vulnerability Scale scores

Classification results	Percentile	Name	Score
Range	Up to 89	High vulnerability	0 to 11
	90+	Low vulnerability	12+

The first analysis adopted score 12 as cut-off point. Discriminant analysis presented MBox = 223.83 *p* < 0.001. λ_wilks_ = 0.39; F_(1, 1752)_ = 2729.13; *p* < 0.001; and canonical correlation = 0.78. This cut-off score managed to properly classify 100% of participants. The second analysis was carried out based on cut-off score 11 (MBox = 78.47 *p* < 0.001. λ_wilks_ = 0.52; F_(1, 1752)_ = 1,613.65; *p* < 0.001; and canonical correlation = 0.69), which managed to properly classify 88.5% of participants. The third test was conducted with cut-off score 10 (MBox = 49.48 *p* < 0.001. λ_wilks_ = 0.65; F_(1, 1752)_ = 915.54; *p* < 0.001; and canonical correlation = 0.58), which managed to properly classify 94.9% of participants. Finally, the last test was conducted with cut-off score 9 (MBox = 31.44 *p* < 0.001. λ_wilks_ = 0.76; F_(1, 1752)_ = 534.48; *p* < 0.001; and canonical correlation = 0.48), which managed to properly classify 97.6% of participants. Based on this scenario, cut-off score 12 was the one presenting the highest accuracy for classification purposes - it was followed by score 9.

Table 5 shoes items’ relative influence on total score. Four (4) items stood out: item “1) Does your health prevent you from doing some daily activities?” (14.16%), item “3) Do you have any illness that requires monitoring?” (16.80%), item “14) Are you monitored by an oral health team?” (15.23%) and item “15) Do you have access to free dental care service?” (17.17%). Results have evidenced significant influence of two dimensions on the overall score, namely: “Health Services” (45.66%) and “General Health” (34.67%). On the other hand, “Infrastructure” and “Oral Health” presented modest contributions to the instrument’s score composition: 11.13% and 8.54%, respectively.

**Table 5:** Items’ relative influence on total score

Dimension	Item	Relative Influence
General Health	Does your health prevent you from performing some daily activities?	14.16
	Do you have any movement impairment?	8.46
	Do you have any disease requiring monitoring?	16.80
Oral Health	Do you consider oral care important?	2.42
	Do you believe that oral diseases can be prevented?	0.00
	Do you believe you treat your oral health in a responsible manner?	2.42
	Do you believe it is important to have all teeth in your mouth?	0.00
Infrastructure	Is there a bathroom in your home?	3.68
	Is there electricity in your home?	1.49
	Is there running water in your home?	4.70
	Is there sewage collection in your home?	4.78
Health Care Services	Do you know the public health center where you can seek medical help?	3.15
	Do you use public health center services?	4.96
	Are you monitored by an oral health team?	15.23
	Do you have access to free dental care service?	17.77

## Discussion

EVO-BR has shown significant potential to be used to assess dental vulnerability in patients treated at PHCU in different Brazilian regions. Collected data analysis enabled developing a score ranging from 0 (indicating higher vulnerability) to 15 (indicating lower vulnerability). This score derived from participants’ responses to the EVO-BR items, and its range enabled classifying individuals into high and low vulnerability ranges. The standardization process reflected score consistency in EVO-BR application and interpretation, as well as evidenced this instrument’s accuracy.

Furthermore, EVO-BR stands out as appropriate tool to stratify individuals based on their dental vulnerability, given its easy use and the solid evidence of its validity [9, 18]. Stratifying implies acknowledging different levels of risk associated with each individual presenting a given oral health issue. Thus, population stratification plays crucial role in organizing the work of health teams, since it allows differentiating users. This process enables planning the care to be provided based on individual needs.

Oral Health Teams in PHC face a complex challenge when it comes to organizing access, given the imbalance observed between high population demand and the limited number of oral health teams available [19]. Despite the expansion in oral health coverage across the country, in recent years, its availability is not enough to guarantee effective access to it. Therefore, although increased oral health coverage has significantly reduced the likelihood of non-access to it, the simple existence of dental services does not automatically imply effective changes in these services’ organization and it results in persistent barriers capable of hindering their use [20].

Currently, access organization is strongly influenced by organizational criteria - be them based on risk or on biological severity - often determined after individual assessment by dental surgeons. However, it is known that disparities in access to health services are overall intrinsically linked to socioeconomic, demographic and organizational factors, and that it can result in less advantageous oral health conditions for historically excluded groups, such as elderly, people with lower schooling and/or purchasing power [21]. Therefore, given the need of implementing population-based management processes, as well as of setting a further systematized organization of oral health care provision, it is imperative incorporating expanded and multidisciplinary tools to help mitigating disparities arising from social disadvantages.

The Brazil is a country of vast territorial dimensions and populous with distinct realities in each region faces the challenge of overcoming the unequal distribution of resources and the planning of effective public health policies to serve the regions of greatest vulnerability. This aims to promote development in these regions and improve the quality of life for their populations. The Northern and Northeast regions are in the range of very high social vulnerability. Meanwhile, the Southeast, Central-West, and South have better rates of social prosperity, reflecting better economic prospects and living conditions in the social environment [22].

In addition, according to the latest National Oral Health Survey of 2010, the oral health of the Brazilian population also shows significant regional disparities, reflecting the country’s socioeconomic inequalities. The study revealed that among young people aged 15 to 19, only 23.9% are free of cavities, and among adults aged 35 to 44, this rate drops to 0.9%, reaching only 0.2% among the elderly aged 65 to 74. These conditions are more pronounced in the North and Northeast regions, which show the worst oral health indicators, highlighting a direct correlation between high social vulnerability and poorer oral health conditions. In contrast, the Southeast, Central-West, and South regions, which have better social prosperity indices, also register better oral health indicators, reflecting greater access to preventive care and dental treatment [23].

Accordingly, one of EVO-BR’s main features lies on its application by any healthcare team professional, rather than being limited to dental surgeon assessment. This fact provides the opportunity for more effective inter-professional performance, since other professionals, such as nurses, nursing technicians, community health agents, among others, can actively participate in the process to identify dental vulnerability, as well as contribute to a more comprehensive care provision process. Furthermore, purely clinical assessments of patients’ health conditions exclusively conducted by dental surgeons do not fully cover individual’s health or allow identifying vulnerable population groups. This factor highlights the importance of adopting instruments to provide a more holistic assessment to individuals by also incorporating their personal perception about both their general and oral health [24, 25].

Relative influence levels ranged between items forming EVO-BR, and it has evidenced factors that can be more relevant and influence this instrument’s performance in specific contexts. Items belonging to the “Health Services” dimension, which were associated with monitoring conducted by oral health teams, as well as with free access to dental treatment, recorded the highest relative influence, altogether. This fact has evidenced the importance of guaranteeing access to oral health services and monitoring provided by Oral Health Teams in PHC [19], since, the presence and effective performance of these teams in specific contexts play significant role in determining individuals’ oral health-vulnerability degree.

Furthermore, the influence of items linked to individuals’ ability to carry out daily activities and to the incidence of diseases requiring monitoring stood out in the “General Health” domain. Nowadays, oral diseases are a relevant public health issue since they contribute to high disease rates, as well as have significant impact on people’s quality of life and, consequently, on their ability to carry out daily activities. They are prevalent diseases capable of causing complications in all age groups - their gradients are mainly differentiated by age and socioeconomic status. They are extremely relevant among adult individuals, since oral conditions are featured by chronic pathologies presenting slow progression [26]. Therefore, the concept of health-related quality of life requires taking into consideration not only factors, such as malaise, pain or functional changes, but also emotional aspects and social functions associated with health [27].

Although the current study was carried out with a convenience sample that is not statistically representative of the Brazilian population, it took into account individuals from all five Brazilian regions. Thus, it covered the country’s wide territorial, cultural and social diversity. However, results have evidenced that the cut-off scores established through the herein performed analyses presented high accuracy in classifying individuals in low or high dental vulnerability ranges.

## Conclusion

Oral Health Teams in PHC face the challenge of organizing access considering the imbalance between high population demand and the number of professionals. Furthermore, access organization occurs based on biological risk determined largely after an assessment with the dentist. At this context, when stratifying dental vulnerability applying EVO-BR scores, it is possible to consider multiple factors. Besides, EVO-BR, can be used by any PHC health professionals, including community health workers, subsiding this way a broad information to prioritizing oral health services offer at PHC context. Thus, EVO-BR has proved to be an important instrument to help identifying oral health determining and conditioning factors in populations served by PHC, contributing to oral health care organization in compliance with universality, comprehensiveness, and equity principles.

Further studies should be conducted to assess the potential applicability of this scale in health professionals’ clinical practice and in public policy planning processes.

## Data Availability

The datasets generated and/or analyzed during the current study are available to the public upon reasonable request to the corresponding author.
